# Lung Cancer Screening: Current Status in the United States

**DOI:** 10.1155/DTE.2.181

**Published:** 1996

**Authors:** Denis A. Cortese

**Affiliations:** Division of Thoracic Diseases Mayo Medical School Mayo Clinic Jacksonville 4500 San Pablo Rd. Jacksonville Florida 32224 USA


*Diagnostic and Therapeutic Endoscopy*, 1996, Vol. 2, pp. 181–183
Reprints available directly from the publisher
Photocopying permitted by license only

© 1996 OPA (Overseas Publishers Association)
Amsterdam B. V. Published in The Netherlands
by Harwood Academic Publishers GmbH
Printed in Singapore

### Lung Cancer Screening: Current Status in the United States

DENIS A. CORTESE

*Division of Thoracic Diseases, Mayo Medical School, Mayo Clinic Jacksonville, Jacksonville, Florida*

*(Received July 3, 1995; in final form December 21, 1995)*

**KEY WORDS:** Sputum cytology, lung cancer, mortality, chest x-ray, lung cancer screening

### INTRODUCTION

Screening with sputum cytology and chest x-ray had no impact on mortality from lung cancer. This was the surprising result of trials of the 1970s and early 1980s sponsored by the National Cancer Institute (NCI). The trials were performed at Johns Hopkins Medical Center, the Memorial Sloan-Kettering Medical Center, and the Mayo Clinic (1–6). The result has generated significant controversy and discussion of the implications of the use of chest x-ray and sputum cytology in clinical practice in the United States.

### NCI-SPONSORED TRIALS

The two NCI-sponsored trials performed at Johns Hopkins and Memorial Sloan-Kettering studied the impact of sputum cytology every 4 months in addition to an annual chest x-ray and compared it with a control group that had only the annual chest x-ray (2–4). The study was designed to determine the effect of sputum cytology on lung cancer. The results at these two centers showed no difference in mortality, strong evidence that sputum is of no additional value to the annual chest x-ray. There was a slight trend for decreased mortality in the squamous cell carcinoma group, but the design of the study was not intended to specifically assess the impact of screening on any particular cell type. Rather, the studies were designed to determine the overall impact of sputum cytology on mortality from lung cancer.

Address for correspondence: Denis A. Cortese, M.D., Division of Thoracic Diseases, Mayo Clinic Jacksonville, 4500 San Pablo Rd., Jacksonville, FL 32224.

The design of the Mayo Clinic study was different (5). This study compared the impact of sputum cytology **and** chest x-ray every 4 months compared with a standard **recommendation** of a yearly sputum test and a chest x-ray. More cancers were identified in the group screened with sputum cytology and chest x-ray every 4 months. More cancers were of early stage and, therefore, more were resected in the screened group. However, there was no difference in the mortality from lung cancer between the two groups (7,8).

From November 1971 through July 1983, a total of 366 incidence cases of lung cancer were identified among the patients enrolled at Mayo. In the group that **received** every 4-month chest x-ray and sputum cytology, there were 206 patients identified with lung cancer for an incidence rate of 5.5/1,000 years. In the control group, the group **advised** to have an annual chest x-ray and sputum cytology, there were 160 patients with lung cancer for an incidence rate of 4.3/1,000 years.

Of the 206 patients in the screened group, 78 (38%) had postsurgical stage I cancer and 5 (2%) had postsurgical stage II cancer. Of the 160 patients in the control group who had lung cancer, 40 (25%) had postsurgical stage I while one had postsurgical stage II disease.

The distribution of cell types in the total 366 incidence cases was fairly even between the screened and the control groups. In the screened group the distribution was: squamous cell carcinoma, 68 (33%); adenocarcinoma, 61 (30%); large cell carcinoma, 29 (14%); and small cell carcinoma, 48 (23%). In the control group the distribution of cell type was: squamous cell carcinoma, 51 (32%); adenocarcinoma, 40 (25%); large cell carcinoma, 24 (15%), and small cell carcinoma, 45 (28%). It was not anticipated that there would be such a high incidence of adenocarcinoma and small cell carcinoma with the proportionate decrease in squamous cell carcinoma. Small cell carcinoma is a disease not amenable to screening because of its rapid progression. As we now know, adenocarcinoma is not easily detected by sputum cytology but when detected by sputum, it is often untreatable.

There was a difference in the number of patients who underwent curative resection between the two groups. In the screened group only 18 patients (9%) had the cancer detected by sputum cytology alone, 15 underwent resection. Seventy-two patients (40%) had the cancer detected by the 4-monthly chest x-ray and 45 of these patients were resectable. Forty-three (21%) patients had the cancer detected by an unplanned nonstudy chest x-ray and 27 still had resectable cancer. Finally, 73 patients (35%) had their cancer detected after they developed symptoms but only 7 cancers were resectable. Of the total 206 patients in the screened group, 94 (46%) had resectable cancer. In the control group of patients, 48 (30%) had their cancer detected by chest x-ray and 36 of these had resectable cancer. Of the patients, 112 (70%) developed symptoms before detection of the cancer and only 15 cancers were resectable. Of the total 160 patients in the control group, 51 (32%) had resectable cancer.

There are at least four important observations from this information. First, only 9% of patients in the screened group had cancer detected by sputum cytology alone. Second, more patients in the screened group underwent resection because of the sputum cytology and the screening chest x-ray. Third, a smaller percentage of patients in the screened group presented with symptoms at the time of detection. Finally, the results in both the study and control groups show that less than 13% of patients have resectable cancers when they present with symptoms (7,8).

In some aspects, screening for lung cancer appears to have been a success. In the study group, there were more lung cancers detected, they were more localized and therefore, more likely to be resected for potential cure. Theoretically, there should, have been a reduction in the mortality from lung cancer in the study group compared to the control group. The surprising finding was that the death rates in the Mayo study were the same. In the four monthly screened group, 122 patients died of lung cancer for a death rate of 3.2/1,000 years. In the control group, 115 patients were identified as dying from lung cancer for a death rate of 3.0/1,000 years. The death rates in both groups related to all causes of death were similar at 24.9/1,000 years and 24.8/1,000 years in the study and the control groups, respectively.

This unexpected result has intrigued physicians for the past 15 years. Possible explanations for the failure of screening to reduce mortality include: the size of the study groups, the statistical power of the study, the distribution of the cell types, overdiagnosis of clinically unimportant cancers, statistical chance, and ineffective therapy.

There were a number of initial assumptions in the original study that proved erroneous. Cytologic examination of the sputum was expected to detect 30 to 33% of all lung cancers, yet only 9% of the patients had cancer detected by sputum cytology alone. A lower percentage of patients had squamous cell carcinoma while a higher percentage of patients had adenocarcinoma and small cell carcinoma than anticipated. It was expected that mortality from lung cancer would not be affected by an annual chest x-ray, yet it appeared that the annual chest x-ray played the biggest role in the detection of these cancers. It was expected that deaths from lung cancer would be easy to determine and identify, but this did not prove to be the case, particularly in the control group. And finally, it was anticipated that the number of lung cancers in the control group would equal the number in the 4-monthly screened group.

The cell type has dramatic prognostic implications. Squamous cell carcinoma and adenocarcinoma, even when roentgenographically occult, behave as different cancers (9). In squamous cell carcinoma, approximately 62% of patients will have positive sputum cytology. Up to 43% of those with abnormal sputum have been reported to be x-ray occult. Squamous cell carcinoma, particularly when x-ray occult, is resectable at a rate above 80%. X-ray occult squamous cell carcinoma has proven to have unexpected nodal disease in approximately 17% of patients (10). The 5-year survival for roentgenographic occult squamous cell carcinoma is more than 90%. Adenocarcinoma is dramatically different (11). In adenocarcinoma, positive sputum is obtained in no more than 25% of the patients. Adenocarcinoma is very rarely roentgenographically occult. When sputum cytology is positive more than 70% of the patients will have nodal disease. The 5-year survival of patients with adenocarcinoma when the sputum cytology is positive is 0%. Small cell carcinoma, of course, is rapidly progressive and when sputum cytology is positive, it is invariably at an advanced stage. Therefore, the distribution of cell type in a patient population will have a great impact on the ability of sputum cytology to reduce mortality.

The possibility of overdiagnosis of lung cancer when it was not clinically significant, was a concern in the Mayo study. However, other studies indicate that lung cancer is usually virulent. In an autopsy series published in 1968 of 3,286 patients, 26 unsuspected lung cancers were found (12). Of these, nodal involvement was found in 57%. Most of the cancers contributed to the patient's death although it was not suspected at the time of death. The conclusion of these authors was that lung cancer was not likely to remain clinically latent for long (12,13).

A study of the survival of patients with stage I lung cancer who underwent surgery, versus a group who did not have surgery, was published in 1992 (14). This study was a review of the NCI-sponsored screening study at all three institutions. There were 336 patients who had stage I lung cancer. Of these patients, 45 did not have surgery for various reasons. The 5-year survival of patients with stage I lung cancer after surgery was 70%. The 5-year survival for patients with stage I lung cancer without surgery was less than 4%. The conclusion was that stage I lung cancer is likely to be progressive and that the 5-year survival was significantly reduced without surgery. Another article reviewing the survival of patients with stage I lung cancer, not surgically resected, comes from Japan (15). This was based on the Japanese screening study between the years 1976 and 1981. The study was performed at 20 institutions. There were 42 screen-detected and 27 sputum-detected patients with stage I lung cancer who did not have surgery. The median survival was 25 months in the patients who had the stage I cancer detected by the screening studies. The survival was only 13 months in the 27 patients who had symptom-detected stage I cancer. Lung cancer mortality in these two groups of patients was 80% in the screen-detected patients and 81% in the symptom-detected patients. Therefore, it appears that overdiagnosis is not a likely explanation since the mortality rate was the same in both groups.

Could random chance explain the lack of difference in mortality? A recent article reassessed the statistics of the Mayo study (16). There was only a 0.98% difference in the lung cancer detection rates: 4.46% in the study group and 3.48% in the control group. If the cancer rates had been the same in the two groups and if the same 85% mortality rate had occurred in the control group, there would have been a mortality advantage of screening at a $P = 0.052$ level. These authors state that the statistical power of the Mayo lung project had a 90% probability to detect a mortality reduction of 50%. However, the study had only a 40% probability to detect a mortality reduction of less than 20% between the two groups.

### CONCLUSIONS

With all this information, the only conclusions that can be drawn from the NCI-sponsored studies are: 1) there are insufficient data to conclude that chest x-rays are of no value in lung cancer screening since all patients in all groups in all three centers received chest x-rays, and 2) there are no definite data that sputum cytology testing adds to the chest x-ray in the screening and treatment for lung cancer. As a result, the NCI is funding a new series of trials in the United States. For these trials, patients (men and women) between the ages of 60 and 74 will be enrolled. The sample size will be 74,000 patients in each group for a total of 148,000 patients. This study is to have a 90% power to detect a reduction in mortality of 10%. These trials are just getting underway; unfortunately we will have to wait many years before the results are known. It is certain that we will learn much about lung cancer during these trials, and we hope it will benefit our patients.

### REFERENCES

1. Berlin NI, Buncher CR, Fontana RS, et al. Early lung cancer detection: introduction. Am Rev Respir Dis 1984;130:545–549.
2. Frost JK, Ball WC, Levin ML, et al. Early lung cancer detection: results of the initial (prevalence) radiologic and cytologic screening in The Johns Hopkins study. Am Rev Respir Dis 1984;130:549–554.
3. Flehinger BJ, Melamed MR, Zaman MB, et al. Early lung cancer detection: results of the initial (prevalence) radiologic and cytologic screening in the Memorial Sloan-Kettering study. Am Rev Respir Dis 1984;130:555–560.
4. Melamed MR, Flehinger BJ, Zaman MB, et al. Screening for early lung cancer: results of the Memorial Sloan-Kettering study in New York. Chest 1984;86:44–53.
5. Fontana RS, Sanderson DR, Taylor WF, et al. Early lung cancer detection: results of the initial (prevalence) radiologic and cytologic screening in the Mayo Clinic Study. Am Rev Respir Dis 1984;130:561–565.
6. Berlin NI, Buncher CR, Fontana RS, et al. Early lung cancer detection: summary and conclusions. Am Rev Respir Dis 1984;130:565–570.
7. Taylor WF, Fontana RS, Uhlenhopp MA, et al. Some results of screening for early lung cancer. Cancer 1981;47:1114–1120.
8. Fontana RS, Sanderson DR, Woolner LB, et al. Lung cancer screening: the Mayo program. J Occup Med 1986;28:746–50.
9. Cortese DA. The prognostic value of sputum cytology. Chest 1992;102:1315–1316.
10. Cortese DA, Pairolero PC, Bergstrahl EJ, et al. Roentgenographically occult lung cancer: a ten-year experience. J Thorac Cardiovasc Surg 1983;86:373–380.
11. Muira H, Konaka C, Kawate N, et al. Sputum cytology-positive, bronchoscopically negative adenocarcinoma of the lung. Chest 1992;102:1328–1332.
12. McFarlane MJ, Feinstine AR, Wells CK. Clinical features of lung cancers discovered as a postmortem "surprise". Chest 1986;90:520–523.
13. Chan CK, Wells CK, McFarlane MJ, et al. More lung cancer but better survival. Implications of secular trends in "necropsy surprise" rates. Chest 1989;96:291–296.
14. Flehinger BJ, Kimmel M, Melamed MR. The effect of surgical treatment on survival from early lung cancer. Chest 1992;101:1013–1018.
15. Sobue T, Suzuki T, Matsuda M, et al. Survival for clinical stage I lung cancer not surgically treated. Cancer 1992;69:685–692.
16. Strauss GM, Gleason RE, Sugarbaker DJ. Screening for lung cancer re-examined. A reinterpretation of the Mayo lung project randomized trial on lung cancer screening. Chest 1993;103:3375–3415.

